# Identifying and addressing health-related social needs: a Medicaid member perspective

**DOI:** 10.1186/s12913-024-11605-9

**Published:** 2024-10-08

**Authors:** Meagan J. Sabatino, Kate Sullivan, Matthew J. Alcusky, Joanne Nicholson

**Affiliations:** 1 Department of Population and Quantitative Health Sciences, University of Massachusetts Chan Medical School, Worcester, MA USA; 2 For Health Consulting, University of Massachusetts Chan Medical School, Worcester, MA USA; 3 The Heller School of Social Policy and Management, Brandeis University, Waltham, MA USA; 4 Department of Psychiatry, University of Massachusetts Chan Medical School, Worcester, MA USA

**Keywords:** Medicaid, Health-related social needs, Accountable care

## Abstract

**Background:**

Multiple state and national health care organizations have invested in activities to screen for and address the health-related social needs (HRSNs) of their patients. However, patient perspectives concerning HRSN screening discussions and facilitated referrals to supports are largely unexplored. The main objectives of this study were to explore the ways in which Massachusetts Medicaid (MassHealth) members engage with their health care clinicians to discuss HRSNs, to identify common needs discussed, and to describe whether members feel these needs are being addressed by health care clinicians and staff.

**Methods:**

The study team performed a cross-sectional, qualitative research study that included in-depth, open-ended interviews with 44 adult MassHealth members. Interviews were conducted between June and October 2022. Interviews were recorded, transcribed, and systematically coded for analysis, and common themes were reported. The data collected for this study were part of a larger independent evaluation of MassHealth’s 2017-2022 Section 1115 Demonstration that granted authority from CMS to implement health care delivery system reforms in Massachusetts.

**Results:**

In this qualitative study of Medicaid members, some reportedly felt comfortable freely discussing all of their clinical and social needs with their health care clinicians, while others noted feelings of apprehension. Several members recalled being asked about their HRSNs in various clinical or community settings, while others did not. The majority of members endorsed having an unmet HRSN, including housing, nutrition, financial, or transportation issues, and many barriers to addressing these HRSNs were discussed. Finally, many members cited a preference for discussing HRSNs with community-based care coordinators and social workers at the community partner organizations rather than with their health care clinicians. Community-based care coordinators were lauded as essential facilitators in making the connection to necessary resources to help address HRSNs.

**Conclusions:**

Study results highlight an opportunity to increase the effectiveness of HRSN screening and referral practices within the health care setting through relationship building between Medicaid members and diverse interdisciplinary care teams that include staff such as community health workers. Continued investment in cross-sector partnerships, screening workflows, and patient-clinician relationships may contribute to establishing an environment in which members can comfortably discuss HRSNs and connect with needed services to improve their health.

**Supplementary Information:**

The online version contains supplementary material available at 10.1186/s12913-024-11605-9.

## Background

Unmet health-related social needs (HRSNs), that is, the economic and social needs of an individual that affect their ability to maintain their health and well-being, arise from the economic and social conditions in which people grow, live, and work [1]. Health and wellness are influenced by social determinants at the individual (e.g., income, education) and structural levels (e.g., structural discrimination, neighborhood environment) [1]. Unmet HRSNs are intersecting, multidimensional circumstances that are associated with higher rates of morbidity, mortality, and disability, higher health care costs, and worsening health disparities [2–8]. While unmet HRSNs negatively affect health, a substantial body of research has highlighted that when HRSNs are addressed, health outcomes improve [5, 7, 8]. Social service interventions targeting homelessness for the chronically ill have been reported to reduce acute and emergency health care utilization, and programs aiming to address food insecurity have been associated with reported decreases in barriers to medication adherence, including cost-related medication underuse [6, 9]. Health care clinicians and organizations have recognized the importance of identifying HRSNs to provide social-risk informed care and social-risk targeted care to improve the health of their patients [3, 4, 7, 9, 10]. 

Multiple states and national health care organizations have recently invested in activities to promote HRSN screening and data collection to identify and understand the needs of patients. In some cases, such activities are incentivized or contractually required; Medicare and Medicaid programs have implemented quality measures to hold clinicians accountable for HRSN screening [11, 12]. The Agency for Healthcare Research and Quality (AHRQ), the Centers for Medicare and Medicaid Services (CMS), and the Centers for Disease Control (CDC) are among many organizations that have invested in HRSN data collection infrastructure, the development of validated HRSN survey instruments, and prediction models to further investigate the role of social determinants of health in the health outcomes of communities [13–17]. 

Compared with Medicare and commercially insured populations, Medicaid enrollees have substantially higher rates of unmet HRSNs contributing to health inequities and poor health [3, 12, 18]. Early in 2021, the Centers for Medicare and Medicaid Services (CMS) released a State Health Official letter encouraging states to identify HRSNs and help Medicaid members address unmet needs [15]. States can address member HRSNs through a variety of Medicaid authorities, including state plans, 1915(c) waivers, managed care in lieu if services, and Sect. 1115 demonstrations. CMS also required that individuals receiving these services under Medicaid authorities have a documented HRSN [15]. Previous studies have shown that patients are generally receptive to being screened for HRSNs; however, some patients are concerned about experiencing discrimination based on their responses to screening questions [19]. Historical injustices and ethics violations that have led to distrust in the health care system may contribute to Medicaid member apprehension with HRSN screening and acceptance of social service supports [19–21]. Additionally, the heterogeneity of HRSN screening processes and social service deployments across organizations may contribute to differential experiences and levels of satisfaction among Medicaid members.

### Study objectives

Despite increasing system-wide investments focused on screening and interventions, Medicaid member perspectives concerning HRSN screening discussions and access to supports are largely unexplored [3]. The main objectives of this study were to explore the ways in which Massachusetts Medicaid and the Children’s Health Insurance Program (MassHealth) members engage with their health care clinicians to discuss HRSNs, to identify common needs discussed, and to describe whether members feel these needs are being addressed by health care clinicians and staff.

## Methods

Given the exploratory nature of this study, semi-structured interviews with MassHealth members were conducted and data obtained were content-analyzed inductively [22]. Data were obtained in the final year of the five-year (2017–2022) 1115 Demonstration Waiver program to capture members’ perspectives following the implementation of HSRN screening procedures and social service deployments to address identified needs.

## Study design

This cross-sectional, qualitative research study included in-depth, open-ended interviews with MassHealth members, conducted between June and October 2022. The interview data collected for this study were part of a larger independent evaluation of MassHealth’s 2017–2022 Sect. 1115 Demonstration Waiver that granted authority from CMS to implement health care delivery system reforms in Massachusetts [23, 24]. Additional details regarding the design and methods for the independent evaluation interviews are described in previous literature [25, 26]. The development of the interview guide (Supplement 1) was informed by a member work group, with modifications made to incorporate member feedback.

The University of Massachusetts Chan Medical School Institutional Review Board (IRB) determined that this study did not meet the criteria for human subjects’ research and ethics approval was not required; however, standard ethical principles and practices were followed, and verbal informed consent was obtained from members to audio record interviews for transcription.

### Conceptual framework

The Consolidated Framework for Implementation Research (CFIR) served as a theoretical framework for the larger evaluation, guiding interviews with key informants and data collection procedures [27]. The CFIR contains a set of constructs, located within five separate domains, associated with the successful implementation of evidence-based practices and is commonly used as a framework for evaluating both facilitators and barriers to implementation efforts [27]. The development of interview protocols and the initial framework for the qualitative analysis of the interview data were guided by the CFIR, as detailed in previous publications.

### Context

In 2017, MassHealth, took steps toward integrating care to address the full spectrum of member needs, including HRSNs, by launching new delivery system and payment reforms. As part of a CMS 1115 Demonstration Waiver, in 2018 MassHealth launched 17 accountable care organizations (ACOs) and 27 partnering community-based organizations (i.e., Community Partners, CPs) serving MassHealth members across the Commonwealth [24]. One of the program goals was to promote, through clinician and organizational incentives, annual screening of HRSNs for all ACO enrollees. Although all ACOs were required to screen for a core set of HRSNs (housing, nutrition, transportation, utilities), MassHealth provided ACOs with the flexibility to choose two additional HRSNs (employment, training or education, experience of violence, or social support), to select their HRSN screening instrument, and to determine the screening process, to tailor these elements to their population’s needs. ACO providers and CP staff used available resources to address HRSNs, including available state and federal social service programs and existing community-based supports. Additional programs were established in later years of the ACO program, including the Flexible Services program launched in 2020, to address identified housing and nutrition needs for a subset of the ACO membership through community-based social service organization (SSO) partnerships [19]. 

### Participating members and recruitment efforts

The study sample consisted of MassHealth adult members receiving care from one of seventeen MassHealth ACOs participating in the MassHealth 1115 Demonstration Waiver. Members were referred through the use of standardized forms, distributed by email to leadership at ACO and community-based “Community Partner” (CP) organizations. The email requested that clinicians and staff at each organization complete a referral form identifying members with complex medical conditions, those receiving behavioral health care, and those receiving long-term services and supports (LTSS), who may be willing to participate in an interview to share their health care experiences. The referral form included information regarding the member’s name, email, phone, preferred method of contact, zip code, ACO or CP serving the member, age, sex, race, ethnicity, gender identity, primary spoken language, medical conditions, and social and clinical support services received. ACO clinicians and CP staff referred a total of 118 MassHealth members. The research team reviewed and contacted all submitted referrals. About 63% of those referred, or a total of 74 members, were not 18 years old or older, could not be reached after three attempts, declined to participate, or did not attend the scheduled interview. A total of 44 MassHealth adult members from 13 ACOs participated in the semi-structured interviews. At the time of the interviews, all ACOs were offering referrals to state and federal supports, connections with community-based supports, and Flexible Services programs to address HRSNs.

### Interview procedures

Using a standardized outreach script, outreach to referred MassHealth members was conducted by experienced researchers by telephone or email, based on preferences noted on the member referral form. Accessibility accommodations (e.g., assistance from a personal care assistant, additional time) were arranged and employed as needed. Interviews were conducted by telephone or videoconference (e.g., Zoom) by experienced interviewers, who were trained and communicated frequently in routine research team meetings to discuss and resolve any questions or issues that arose in the interview process. All interviews were conducted in the preferred language of the member with the use of interpreter services, as needed. Members were provided with an interview fact sheet, in the member’s primary spoken language, within the three days prior to the scheduled interview appointment. All members gave verbal consent upon initiation of the interview protocol and consent for audio recording. Though written informed consent was not required, the study was explained to MassHealth members, who were told that participation was voluntary. Members were given ample opportunity to ask questions about the interview processes and procedures and were told they had the right to withdraw from the interview at any time.

The interview guide was developed in collaboration with expert consultants and a Member Work Group, inclusive of member and member advocates, to advise on member engagement processes and procedures. The interview guide consisted of twenty-eight questions, informed by the Outer Setting(features of external context or environment that might influence organizational actions) and Inner Setting (features if implementing organization that might influence implementation) domains of the Consolidated Framework for Implementation (CFIR) covering a variety of topics such as health care goals, visits with primary care clinicians and specialists, and experience using telehealth services [27]. Use of telehealth was particularly relevant as interviews were conducted subsequent to the COVID-19 pandemic and the concomitant increase in telehealth service provisions [28]. 

Data were collected regarding general health, care-seeking behaviors, and accessibility of care (e.g., length of time waiting for an appointment). Additionally, questions such as, “Did you talk with your health care clinician about other needs that you may have beyond your health like housing, transportation, or nutrition,” “What kind of needs did you talk to them about,” and “Did they help you address these needs?” were discussed. Members were provided a $50 gift card for their time and effort in participating in the interview process. All interviews were performed over telephone or Zoom and lasted approximately 60 min. With member consent, all interviews were audio recorded, and then deidentified, translated, and transcribed for qualitative analysis.

### Data analysis

The codebook and subsequent analysis were informed by review of the CFIR framework [27]. In addition, emerging themes were identified by the qualitative working group, comprised of trained and experienced analysts. A coding schematic was developed by the working group after reviewing two to three transcripts and refined through an iterative process in subsequent group discussions. Through a process of concurrent coding of a subset of interviews, comparisons of coding approach, and refinement of the coding schematic, a shared understanding of codes and agreement among coders was established. Using a weighted Cohen’s Kappa, transcript coders were verified to be in almost perfect agreement (0.81-1.00) [29]. Transcripts were then randomly assigned to group members for independent coding. Throughout the coding process, the qualitative working group met weekly to discuss and define emerging themes to update the coding schematic, as needed. Coded transcript data were managed using Dedoose [30]. Following the completion of initial coding, a memo writing process was employed [31]. Memos were used to summarize the coded data, to identify and specify thematic patterns within and across interviews. Memos were discussed by the qualitative working group to ensure agreement on reported themes and patterns in members’ experiences.

## Results

### Participating members

The study sample consisted of 44 adult MassHealth members, ages 27 to 64 years, of whom 27 (61%) identified as women. 73% of those interviewed reported having a complex medical condition (e.g., diabetes, asthma, obesity, heart disease, etc.), 68% reported receiving behavioral health (BH) services for various conditions (e.g., anxiety, depression, post-traumatic stress disorder (PTSD)), and 34% reported receiving LTSS supports such as personal care services to assist with activities of daily living. Around 20% of members interviewed reported receiving both BH and LTSS services, and 61% were enrolled with a Community Partner organization receiving care coordination services. At the time of the referral for interview participation, 52% of members self-reported a current HRSN, and 50% of the members interviewed were already receiving housing supports, nutrition supports, or both. Additional member characteristics are described in Table 1.

**Table 1:** Participating member demographics and characteristics

Participating Member Demographics and Characteristics (*n* = 44)	
**Age**	**Mean (Range)**
Interviewed Members	49 (27–64)
**Sex Assigned at Birth**	**n (%)**
Female	28 (64%)
Male/Unknown^a^	16 (36%)
**Gender Identity**	**n (%)**
Female	27 (61%)
Male/Other/Unknown^a^	17 (39%)
**Race**	**n (%)**
White/Caucasian	22 (50%)
Black or African-American/Other/Unknown^a^	22 (50%)
**Ethnicity**	**n (%)**
Non-Hispanic	30 (70%)
Hispanic/Unknown^a^	14 (30%)
**Primary Spoken Language**	**n (%)**
English^a^	*≥* 33 (75%)
Other/Unknown^a^	*≤* 11 (25%)
**Services Received**	**n (%)**
Behavioral Health	30 (68%)
Long-Term Services and Supports	15 (34%)

^a^Categories appear combined or cell sizes are non-specific to mask the number for fields and complimentary field where cell sizes could be < 11.

### Members’ experiences with HRSN screening and services

In this sample of MassHealth members, three key themes were identified: (1) communication with health care clinicians and staff regarding HRSNs; (2) how identified HRSNs were being addressed; and (3) barriers to addressing HRSNs. Each theme was summarized, with illustrative quotes to support theme interpretations provided in Table 2.

**Table 2:** Representative member quotes

Representative Member Quotes	
**Theme**	**Representative Quotes**
**Communication Regarding HRSNs**	“I feel pretty comfortable talking with my doctor about my needs. Like maybe 80%. I don’t know her that well yet.”
	“I probably wouldn’t talk to my provider about my social needs… I think that just comes from maybe my generation or something, or just the way overall people look at things like that. All the stuff that comes with, ‘you shouldn’t need that,’ or ‘you shouldn’t apply for that.’ ‘You’re freeloading’… I struggle with actually asking for help.”
	“With my care coordinator, I met with her before COVID. Her and another therapist met me at [a coffee shop], and we talked for about an hour and she’s been my care coordinator ever since.”
**How Unmet HRSNs are Being Addressed**	“My care coordinator has taken me shopping to get groceries, she’s taken me for the vaccine, when I didn’t have a ride to Boston, twice.”
	“For transportation, because of the back surgeries that I have, my doctor filled out the handicap placard thing for me and helped me to apply for [the transportation service]. My doctor also told me about [another transportation] pilot program, which I enrolled in.”
	“My care coordinator schedules my appointments, she helps me get rides, she helps me with housing, and paperwork… like it’s crazy. I never met someone that does that.”
**Barriers to Addressing HRSNs**	“I live $3,000 below the poverty line. For an individual, 13,500 is the poverty line. So, you see what the struggle is…And from the time of the month, where people have less food because the food stamps don’t come until next week, they’re calling food pantries. And they’re going to the food pantries, if they have a car.”
	“There’s been times that I’ve gone to the hospital, and they want to sit there and then discharge me at maybe two or three o’clock in the morning. I can’t call an [ride share application] and public transportation stops running at a certain time.”
	“The case worker did reach out to me, but that’s because we were still trying to get into the family shelter at that point… I just had a baby…I needed a bank statement to get into the family shelter.”
	“When I call the food pantry, you have to redial the local number until you get through. Sometimes it takes you over 100 calls, redialing to finally get through. And since COVID, they let you get food every two weeks…You get a busy signal, and you get a “please try back later,” then finally, it could be 150 tries, because everybody in the city’s trying to call at the same time. They don’t have a hotline with different lines. Pick up for everybody is at once, and you can only call between 10:00 and 12:00. If you don’t get through by 11:30, all the orders are taken, you go without food.”
	“I was in prison and I worked in the optical shop, so I made glasses. I know my prescription, I know what to look for, so I usually just go online and spend twenty bucks, thirty bucks and get a pair of glasses. It is quite a skillset, except nobody wants to hire a felon.”

### Communication regarding HRSNs

Overwhelmingly, members shared that they had discussions regarding HRSNs with health care workers in a variety of settings. Several participating members recalled being specifically asked about their HRSNs and others did not. A few members stated they were specifically asked about singular needs, such as transportation or food insufficiency, but did not participate in what they would have perceived to be a formal screening. The specific health care workers that members identified as implementing screening processes included clinicians, such as doctors and advanced practice clinicians, behavioral health workers, counselors, care coordinators, case workers, member advocates, and member navigators. The HRSN discussions took place in a variety of settings, including within a clinic, via telemedicine, and in community settings, including coffee shops or other public spaces. HRSN discussions that took place in public spaces were likely to be with care coordinators, case workers, and counselors.

Members varied in their level of comfort in speaking with their health care clinicians about their HRSNs. Some members reported feeling comfortable discussing any issue with their clinician, especially if they had established a positive rapport with the clinician and found them to be helpful in meeting their health care needs. Some members stated they would not feel comfortable talking about these issues with their clinicians because they may be perceived as trying to “freeload,” or because they did not feel their health care clinician was the best resource to address their social needs. Many members cited preference for discussing HRSNs with community-based care coordinators and social workers at the CP organizations rather than with their health care clinicians.

### Addressing identified HRSNs

Several interviewed members described themselves as having a number of HRSNs that were being met through a variety of resources. While a few members stated they had spoken to their health care clinician about their needs, without solicitation, and did not feel the clinician took action to support them, many members indicated they were referred by clinicians and staff to community-based organizations to meet their needs. MassHealth CPs and SSOs were highly-cited as resources that coordinated or delivered services to meet member HRSNs. When services to address unmet HRSNs were obtained through the support of health care clinicians, the connection to services was most often coordinated by care coordinators and office support staff. Community-based care coordinators were lauded as essential facilitators to make the connection to necessary resources. A handful of members reportedly relied on family, friends, and neighbors to address their needs, while others stated they were able to set up services on their own. Finally, the Sheriff’s office was a cited resource for members who were recently incarcerated, helping them to obtain clothing and to be connected with social service supports to meet their needs.

Transportation issues were the most cited unmet HRSN. Members received transportation supports through programs such as MassHealth’s Prescription for Transportation (PT1) program, public transportation, the Uber Mass Pilot Program, and directly from their care coordinators. With respect to food insecurity, meal delivery services and grocery gift cards were cited as helpful aids to obtain food from the perspective of members. Moreover, some described that they obtained primary food sources, or supplementing food received through meal delivery or grocery gift cards, from local food pantries. Housing instability and homelessness were described by more than half of the members, with shelters, housing vouchers, help from others in looking for apartments, rental assistance, agency housing supports, and coverage of utility costs helping to meet their needs. Lastly, legal support, help with job seeking, and clothing were also cited as needs that were being met through a variety of social services.

### Barriers to addressing HRSNs

Barriers to addressing HRSNs were commonly reported among our sample of Medicaid ACO enrollees interviewed in 2022, four years after the ACOs were launched and new HRSN screening requirements went into effect, and two years after new resources were made available to ACOs for addressing housing and nutritional HRSNs. Multiple members noted barriers to obtaining transportation throughout the interviews, reporting that they could not rely on public transportation as it was often unpredictable, was not available in evening hours, and required members to take multiple different public transportation modalities to reach their destination, a time-consuming process. Ride-sharing apps were acceptable options, but vouchers were often not readily available and these services were reported as not consistently having accessibility accommodations, such as wheelchair or child seat straps. A portion of members stated they needed to pre-request certain covered transportation services 24 to 48 h in advance, limiting their ability to attend same-day or emergency outpatient appointments. This issue, coupled with clinicians who were not always willing to accommodate telemedicine appointments, led some members to choose their health care clinicians based on nearest location and telehealth availability, restricting options for care.

In members with food insecurity, transportation limitations also restricted their ability to get to food pantries or grocery stores. This was especially a concern of members located in self-described “food deserts.” Food pantries, some of which only took phone orders following the onset of the pandemic, were reported to run out of supplies throughout the day. Members, who wanted to ensure their orders were received by the food pantries before food supplies were depleted, had to continually redial the food pantry phone number for hours at a time on the day of food release because the pantries did not have a phone tree or call waiting system. While reported to be somewhat helpful, the supplemental nutrition assistance program (SNAP, i.e., “food stamps”) did not allow for preferred food options. In addition, one member noted that nutritious options were difficult to obtain, for example, due to the cost of produce.

Financial insecurity affected all aspects of members’ lives. Members reported they couldn’t get eyeglasses, obtain needed surgery, buy quality foods, afford transportation, obtain a gym membership to get healthy, afford phone or internet service to attend telemedicine appointments (which was especially an issue for care access throughout the pandemic), or afford recreational physical activities (e.g., swimming lessons or dance classes) for their children to manage childhood obesity, due to financial insecurity.

Several barriers to obtaining housing services and supports were described. A few members reported difficulty proving they were experiencing housing instability, which is necessary to qualify for some services. Members described needing bank statements to show they didn’t have the financial ability to afford housing but didn’t have a bank account, while others stated they were previously housed in an adult shelter, but had difficulty obtaining housing in a family shelter when they had children. Multiple additional issues including unsafe living conditions, high housing costs, long waiting lists, and limited availability were cited to be problematic in meeting members’ housing needs. Members with physical disabilities had greater difficulty finding housing because not all housing options were handicap accessible. Stable housing was also noted as essential to obtaining other services, such as food delivery.

Members who reported that they were formerly incarcerated referred to their experiences as exceptionally challenging. Specifically, members noted that many in the community didn’t want to hire people with felony convictions, limiting their ability to make a living wage. Others noted that their mental and physical health markedly declined while incarcerated, citing ineffective and low-quality health care received at that time, undermining their physical and mental functioning, leading to the rapid increase in the burden of unmet HRSNs.

Finally, clinical shutdowns, a depleted workforce, and a swift rise of unmet social needs precipitated by the COVID-19 pandemic were generally echoed as barriers to meeting HRSNs by a large majority of interviewed members.

## Discussion

In this qualitative study of Medicaid members, some reportedly felt comfortable freely discussing all of their clinical and social needs with their health care clinicians, while others noted feelings of apprehension. Several members recalled being asked about their HRSNs in various clinical or community settings, while others did not, and the majority of interviewed members endorsed having an unmet HRSN. The HRSNs discussed included a range of needs such as a housing, nutrition, financial, or transportation issues, and many barriers to addressing these HRSNs were highlighted. Members continued to face barriers to addressing HRSNs despite the increased focus on identifying and addressing HRSNs from health systems and their partners, bolstered by dedicated resources invested by the Medicaid program in the years preceding our study. When services to address these unmet HRSNs were obtained as a result of health system screening and referrals, community-based care coordinators were lauded as essential facilitators to make the connection to necessary resources. These data support the notion that health care policies incentivizing or requiring screening for HRSNs are necessary but insufficient; policy makers should consider community contexts and invest in creating environments that enable members to comfortably discuss their HRSNs while equipping their care team with the skills, relationships, and resources to help address them [31–34]. 

Medicaid enrollees have high rates of unmet HRSNs, and our study results are consistent with these findings [18, 35]. A majority of the interviewed members endorsed having a considerable burden of housing, nutrition, financial, and/or transportation needs. It is reported that as much as 50% of the variation in health outcomes, approximately 50% of the cost structure of U.S. public health insurance programs, and a substantial portion of health disparities are related to unmet HRSNs [1, 36]. Many health care organizations are directing their focus toward reducing downstream health disparities by screening for and addressing unmet HRSNs [32, 37]. Under new Joint Commission accreditation requirements that took effect on January 1, 2023 and are aimed at reducing health care disparities, accreditted health care organizations are now required to screen for HRSNs and to provide information about community resources and support services to patients treated at their facilities [17]. Additionally, the Centers for Medicare and Medicaid Services continues to highlight the negative impacts of unmet HRSNs on health outcomes for Medicaid members [15]. States are encouraged to test various evidence-based interventions to screen and address HRSNs under Sect. 1115 Demonstration Wavier flexibilities [15].

The apprehension towards discussing HRSNs with health care clinicians expressed by some members highlights the importance of developing trusting relationships between members and their care team, which may be facilitated by the ongoing shift to alternative payment models. Alternative payment models (APMs) such as ACOs have been identified as attractive alternatives to traditional fee-for-service (FFS) models because clinician organizations are incentivized, through quality and cost benchmarks, to improve health outcomes and manage spending [38]. When such incentives are aligned between an ACO and its clinicians (e.g., using primary care sub-capitation), there is greater flexibility to develop diverse care teams (e.g., involving social workers and community health workers) and deliver services and supports in a manner that is most appropriate for a particular person and circumstance. With less dependence on volume for revenue, care team members may be free to spend more time with members with complex health and social needs to develop trusting relationships conducive to discussing HRSNs [15, 17, 23, 32, 37]. Moreover, members in our study described feeling more comfortable discussing HRSNs with community health workers and social workers at CP organizations then with the health care clinicians in the primary-care setting. Yet responsibility for screening of ACO members often falls on primary care clinicians, highlighting the value of broadening care team composition and expertise to include staff from community-based organizations when appropriate, to screen for and address HRSNs. Quality measures used for accountability purposes should also accommodate such interorganizational relationships to promote screening in a member’s preferred setting and reduce the burden of multiple screenings.

Cross-sector partnerships between health care clinicians and community-based organizations to identify and address HRSNs have the potential to generate improved health care quality and outcomes for Medicaid members [38–41]. In this study, MassHealth members frequently cited community-based organizations (e.g., CPs and SSOs) as providing and facilitating access to essential resources to address their unmet HRSNs. Recognizing the value that these community-based organizations bring to the care continuum for Masshealth members with complex health and social needs, MassHealth has taken steps to formalize and institutionalize partnerships between health care and community-based organizations [24]. A distinguishing feature of the 1115 Demonstration Waiver in Massachusetts was the requirement for ACOs to partner with community-based organizations for care coordination supports [24]. In addition to supporting clinical care coordination, community-based organizations are likely to have the training and expertise to provide culturally-compentent supports, making these partnerships ideal for addressing HRSNs [42, 43]. Studies show that partnerships between health and community-based organizations may help to reduce health care costs, a significant concern for Medicaid programs with high rates of costly complex health and social needs [26, 44]. 

### Limitations/strengths

This study has limitations that should be noted. Due to the nature of qualitative research, our findings reflect the synthesis of detailed information from a sample of MassHealth members, but results are not necessarily generalizable to the larger Medicaid population. Purposeful sampling strategies were employed to ensure a diverse participant sample with a mix of experiences accessing services and health conditions was achieved. It is not known how recently members had a healthcare encounter or when the last HRSN screening occurred, possibly affecting interview responses. All interviews are subject to recall and reporting biases. Nevertheless, our semi-structured interviews were performed by trained personnel to limit these biases. Interviews were performed over telephone or teleconference, which may affect the generalizability of results to Medicaid members without access to such techologies and who may be experiencing greater hardships. Overall, this study is one of the first to provide qualitative assessments by Medicaid members of HRSN screening, services, and supports provided by health care clinicians and staff, and the staff of partnering community-based organizations. Our findings of Medicaid member-reported facilitators and barriers to effectively identifying and addressing HRSNs can guide policymaking and program implementation

## Conclusion

In this qualitative study of Medicaid members, we observed that many individuals remembered being screened for HRSNs, yet some did not feel comfortable discussing their unmet needs with their health care clinicians. The majority of interviewed members endorsed having one or more unmet HRSNs, with community-based organization care coordinators highlighted as essential facilitators to connect with social services. Study results highlight an opportunity to increase the effectiveness of HRSN screening and referral practices within the health care setting through relationship building between Medicaid members and diverse interdiscplinary care teams that include staff such as community health workers. Continued investment in cross-sector partnerships, screening workflows, and patient-clinician relationships may contribute to establishing an environment in which members can comfortably discuss HRSNs and obtain needed services to improve their health.

## Supplementary Information

Supplementary Material 1.

## Data Availability

No datasets were generated or analysed during the current study.

## Abbreviations

ACOs: Accountable Care Organizations
BH: Behavioral Health
CFIR: Consolidated Framework for Implementation Research
CMS: Centers for Medicare and Medicaid Services
CPs: Community Partners
HRSNs: Health-Related Social Needs
IRB: Institutional Review Board
LTSS: Long-Term Services and Supports
MassHealth: Massachusetts Medicaid and the Children’s Health Insurance Program
SSO: Social Service Organization
