# Supplementary material for: Identifying and addressing health-related social needs: a Medicaid member perspective
Source: BMC Health Serv Res. 2024 Oct 8;24:1203. doi: 10.1186/s12913-024-11605-9 (PMC11460098; doi:10.1186/s12913-024-11605-9)
Supplement: Supplementary file 1 — Supplementary Material 1. [file 12913_2024_11605_MOESM1_ESM.pdf]

MEMBER EXPERIENCE INTERVIEW GUIDE:

INTRODUCTION:

[Introduce Self]

Thank you so much for agreeing to talk to me today about your experience as a MassHealth Member enrolled in [ACO organization, if known]. [ACO organization] is one of the 17 MassHealth Accountable Care Organizations, or ACOs, which were created about a few years ago to improve the way your healthcare is delivered. An ACO is a group of healthcare providers that work together as a team to provide care to patients. I'm part of the team that is evaluating the MassHealth ACO programs. In this part of our project, we want to learn more about member experiences receiving health care and with MassHealth. What we learn from these interviews will be reported in our evaluation report.

Did you have a chance to review the fact sheet we sent ahead of time?

[If yes] Great. We want to remind you that we would like to record this interview to accurately capture the information you provide. We would also like to remind you that this interview is completely voluntarily. You do not have to answer any questions you do not want to, and you can end the interview at any time. After all of our interviews, we will combine the information together into a report. Your individual answers and personal information will not appear in the summary report. We will not use personal information like names, addresses, birth dates or dates that you received medical care in the report. No one will be able to identify you by reading the report. Also, we will not share your specific interview responses to MassHealth, or to your healthcare provider(s).

However, records related to this project are considered public record, and we would share this information only if required by Massachusetts law. When we store the records, your name isn't connected with your responses and the interview recording will be transcribed and stored in a de-identified format.

[If no, offer the fact sheet to the interviewee and read the script above without "As a reminder" lead]

[Note to Interviewer: Please remind interviewee that they should not provide any identifying details such as dates of care received, names of doctors, or other identifying information. Stating provider type is acceptable]

Do I have your permission to record?

Turn Recorder On

The recording is now on. Do I still have your permission to proceed with recording?

Do you have any questions before we start?

GENERAL OPENING QUESTIONS:

1. How would you describe your health in general right now? - excellent, good, fair, poor? Why do you say that?
2. How does your health affect your everyday life?
3. About how long have you had MassHealth insurance?
4. Does anyone help you decide what kind of health care you need or what kind of healthcare providers that you need to see? If so, who helps you?
5. Can you name all of the key people that help you with your healthcare needs? (Nurses, Doctors, Care Coordinators, etc.) (Reminder: no names or identifying information)
    a. Probe for their role, such as a nurse, specialist, BH provider, care coordinator?
    b. Probe for frequency of contacts?
6. Where do you go for your regular health care needs such as annual visits, minor injuries, colds, sore throats, ear pain, and anything non-emergency? Probe to see if a primary care doctor or a specialist. (Reminder: no names or identifying information)
    a. How long have you been seeing them/going there?

PRIMARY CARE PROVIDER:

7. (Depending on previous answer, ask: Do you have a primary care provider? A primary care provider is someone that works in a medical office that you would see for checkups) Tell me about the last time you saw your primary care provider or the doctor you see most often:
    a. How long ago was it? (Virtual visits included)
    b. Did you go to this provider because you had a healthcare issue or was it for a regular checkup?
    c. How comfortable were you asking them questions? What kinds of questions did you ask them? Give an example or two of the questions you asked.
    d. Did they give you any advice or recommend anything to you to improve your health? If so, what were the recommendations?
    e. Were there any additional tests, such as lab work or x-rays, that were recommended? Did they help you schedule? Please explain how they helped.
    f. Did they recommend that you see an additional healthcare provider for your health needs? Did they help you schedule it? Please explain how they helped.
    g. Did you talk with your healthcare provider about other needs that you may have beyond your health like housing, transportation, or nutrition?

        [If yes] (Please share what you are comfortable sharing) What kind of needs did you talk to them about? Did they help you address these needs? How?
    h. Was there anything different about this visit compared to visits in the past?
    i. Have you noticed any changes in your care over the past few years?
        a. Has receiving care become easier or more difficult, more or less confusing, or complicated? How so?

   j. Overall, how would you describe your experience with your provider? Would you recommend your healthcare provider to a friend or loved-one?
   k. Do you have suggestions to make your most recent experience any better?

SPECIALISTS:

8. Next, we are going to ask about your experience with specialists. A specialist is a type of healthcare provider that helps you with a specific illness or healthcare problem (Examples include cardiologist for people with heart problems, psychiatrist for mental health conditions, or dermatologist for someone with skin conditions). Do you see a specialist? What kind of specialist provider are you seeing? (Ask questions below for selected specialists)
   a. How long ago was it? (Virtual visits included)
   b. Did you go to this provider because you had a healthcare issue or was it for a regular checkup?
   c. How comfortable were you asking them questions? What kinds of questions did you ask them? Give an example or two of the questions you asked.
   d. Did they give you any advice or recommend anything to you to improve your health? If so, what were the recommendations?
   e. Were there any additional tests, such as lab work or x-rays, that were recommended? Did they help you schedule? Please explain how they helped.
   f. Did they recommend that you see an additional healthcare provider for your health needs? Did they help you schedule it? Please explain how they helped.
   g. Did you talk with your healthcare provider about other needs that you may have beyond your health like housing, transportation, or nutrition?
      [If yes] (Please share what you are comfortable sharing) What kind of needs did you talk to them about? Did they help you address these needs? How?
   h. Was there anything different about this visit compared to visits in the past?
   i. Have you noticed any changes in your care over the past few years?
      a. Has receiving care become easier or more difficult, confusing, or complicated? How so?
   j. Overall, how would you describe your experience with your provider? Would you recommend your healthcare provider to a friend or loved-one?
   k. Do you have suggestions to make your most recent experience any better?

TELEHEALTH:

9. Has anything changed about the care you received because of the pandemic? If yes, please describe these changes.
10. How have you been getting care over the last year? Has this been in the doctor's office, at home or in-person somewhere else, or via the phone or video?
    a. If you have had telephone or video doctor's appointments:
       i. How often were they?

        ii. What platform(s) do you use to connect with your doctors/providers? (Zoom, Skype, FaceTime, WhatsApp, etc.)

   b. Have you had any problems using telehealth, for example with internet access, having the right equipment like a smartphone, difficulty navigating the specific platform used by a provider?
   c. Did meeting on the phone or video conference raise any concerns for you? (i.e., concerns about confidentiality, security, safety)
      i. If so, what kinds?
      ii. What created these concerns? (i.e., setting, privacy, sense of detachment)
   d. Do you prefer one way of getting care over another (i.e., in-person versus telephone vs video) overall or for specific types of appointments?
   e. Are you given the choice of whether to see your doctor/provider in person or having a telehealth appointment?
      i. Which other providers, if any, would you want to see via telehealth that you aren't seeing that way now?
      ii. If needed, are you able to go to the office/hospital (maybe for lab tests or vaccinations)?
   f. Do phone or video visits meet your definition of a "good care visit"? Are you satisfied with them?
   g. Have you continued to see any of your healthcare providers and are you getting the care you need?
      i. Do you think you are getting the care you need through both in-person and telehealth visits? Any differences between the visits that you have noticed?
      ii. [If yes] Any trouble getting an appointment with your provider? Any other troubles with in-person visits that you experienced? (i.e., transportation)
      iii. [If they aren't going into the providers office] Are there other ways you are in touch with your healthcare providers, such as by telephone, video call, messaging (e.g., through a patient portal or through emails/texts from your provider's office)?

HOSPITAL VISITS:

11. Have you been to the emergency room **in the past year**?
    If no, skip to the next question
    If yes,
    a. Thinking about your most recent trip to the emergency room, what brought you there? In a few words, how would you describe your experience?
    b. Were you or the emergency room providers talking with your primary care doctor or other providers you see outside the emergency department while you were there?
    c. Thinking about your most recent trip to the emergency room, what was the process like when you were being sent home? Did you know who to follow-up with, if anyone?

   d. Were you offered any health-related follow-up appointments or helpful services after you were sent home? What services did you choose/receive?
   e. When you were sent home from the hospital, did anyone contact you shortly after to see how you were doing? If so, who?

12. Have you stayed overnight in the hospital **in the past year**?
    If no, skip to the next question
    If yes,
    f. For your most recent overnight stay, what brought you to the hospital?
    a. Were you or the hospital providers talking with your primary care doctor or other providers you see outside the hospital while you were staying there?
    b. What was the process like when you were being sent home? Did you know who to follow-up with, if anyone?
    c. Were you offered any health-related follow-up appointments or helpful services after you were sent home? What services did you choose/receive?
    d. When you were sent home from the hospital, did anyone contact you shortly after to see how you were doing? If so, who?

COMMUNITY PARTNER & FLEXIBLE SERVICES:

13. Now we are going to talk about Care Coordinators and Case Managers. Do you have one or more Care Coordinators or Case Managers? (Someone that helps you get all of your healthcare needs addressed but isn't a doctor or healthcare provider. They may help you get your appointments scheduled, check in with you to make sure you can get to your appointments, etc.)
    a. How did you get connected with them? Did someone refer you? Who?
       i. Do you know where they work?
       ii. If you have multiple care coordinators, do they work together?
14. Did you have to fill out any paperwork, go through any visits, or do anything special to begin working with your care coordinator?
    a. Would you say it was an easy process to begin working with your care coordinator? Why or why not?
15. Tell me what your care coordinator helps you with?
    a. How long have you been working with them?
    b. What do you talk about?
    c. How often do you connect with them?
    d. Do they help you with other things beyond your healthcare needs such as housing, transportation, or nutrition? (Share if you are comfortable)
    e. What types of things do they help you with that isn't healthcare related?
    f. How do they go about helping you with these needs? Describe.
    g. [If not already addressed] Do your providers and care coordinators work closely together [as a care team]? If you have a visit with one, are others present, and if not will they be up to date on what happened at your other visits next time you see them?

   h. If you are admitted to the Emergency Department (ED), does your care coordinator help you with planning for after you are discharged?

16. Has your overall health changed now that you are working with your care coordinator? If so, how has it changed?
17. Has a health care provider connected you to an organization to provide you with specific housing services or goods, like assistance with finding housing, or a specific food-related services of goods, like medically tailored meals or vouchers? This is called Flexible Services.
17. If you are receiving Flexible Services, what has that experience been like for you?
    a. Can you tell me which service or services you enrolled in?
    b. How did you find out about these services? Did you find the process of agreeing to Flex easy? Anything that might make it easier?
    c. Once you agreed to services, were you able to receive them quickly? If not, why?
    d. How has your experience with these services been?
    e. If you received housing services or goods, how have these services changed (if at all) your housing situation?
    f. If you receive nutrition services or goods, how have these services impacted your nutrition status?
    g. Are you happy with the services you received? Why/Why not?
    h. Have you seen improvement in your health since you received these services?
    i. Do you have any suggestions for changes?
    j. Would you recommend these services to a friend or loved-one if they needed the same service? Why?
    k. Are there any other services that you wish were available?

BH AND LTSS SERVICES:

18. We would like to learn more about how Behavioral Health and/or Long-Term Services and Supports may or may not have impacted your overall health. Behavioral Health Services help members with needs related to mental health conditions and substance use disorders. Long-term Services and Supports includes a wide range of help provided to people with certain disabilities or chronic health conditions, to allow these people to live more independently. This may include assistance with personal needs such as bathing and grooming, healthcare needs such as managing medication, and activities of daily living such as cooking or driving.
    a. What information has your care coordinator shared with you about the types of services, like these, you could receive
    b. Has your care coordinator helped to connect you to any services which help you with these needs?
    c. Do you receive any services like these?
    d. How has your experience with these services been?
    e. Would you recommend these services to a friend or loved-one if they needed the same service?
    f. Do you have any suggestions for changes?

<u>HEALTH CARE GOALS:</u>

19. I want to hear more about how you set health-related goals for yourself and how you make decisions about what treatment you would like to receive.
    a. How do you decide what is the most important health goal for yourself?
    b. If you have a Care Coordinator, do they work with your healthcare providers to identify goals that you need to work on for your health?
    c. How do you communicate with your healthcare providers or care coordinator what goals are most important to you? Do your healthcare providers or your care coordinator help you decide which ones you should focus on? Do you feel your healthcare providers or care coordinator support you in deciding the goals that you want to work on?
    d. Are there specific providers within your care team you feel more or less comfortable talking to?
20. The goals you set with your providers are usually written down and referred to as a Care Plan. You may have signed this Care Plan. You may have had a discussion with your Care Coordinator or Healthcare Provider about the goals you would work on. I'd like to ask you some questions about your Care Plan.
    a. Do you remember having a care plan?
        i. If yes: Do you have a copy of your care plan?
        ii. Do you know what is included in your care plan?
        iii. Can you tell me about some of your health-related goals?
        iv. Do you have non-medical goals as part of your care plan?
        v. Do you know who was involved in putting your care plan together? Did you and your care team agree on what goals should be included?
        vi. How are your healthcare providers and care coordinators working with you to meet your goals?
        vii. If you have questions related to your health-related goals, do you know who you should go to with these questions?
        viii. Does anything get in the way of meeting your goals? If so, what types of things?
    b. Do you have anything in your health that has improved that you'd like to share? How did your Care Coordinator and/or healthcare providers help you accomplish this?
    c. Do you have anything else that you think impacts your overall health but are not currently addressing? Would you like to address this?
21. Can you talk about your experiences with getting the care/services you want or need in a timely manner?
    a. Do you experience any delays in getting care? If so, please explain.
    b. Is there any care you are <u>not</u> getting that you want/need? If so, why do you think you aren't receiving the care? (Probe for availability/accessibility, disability, language)
    c. Do you feel your healthcare providers and care coordinators understand your medical needs? Please explain why or why not.

22. Can you tell me how your healthcare providers and/or care coordinator help you understand all of the care that might be available to you through MassHealth?
    a. What kind of information do they provide to you about the services that you can take advantage of?
        i. What type of information is really helpful for you? What type of information is not helpful for you?
        ii. Where/how else are you finding this information if it is not being shared with you?
    b. How would you like to receive information from your care team? (i.e., electronic, paper, text, phone call)
    c. Is there any information you would like to receive, that you aren't currently receiving? If so, what would that include?

CLOSING QUESTIONS:

23. What factors are most important to you in selecting a provider or care coordinator?
24. For my next question, let me start with some examples. Sometimes, people want to have a provider who is similar to them in some way, such as: if someone is Hispanic, that person might have a preference for a healthcare provider who is also Hispanic; or someone who is gay may want a healthcare provider who is gay.
    a. Have you had a health care provider or care coordinator who was different from your preference? Was that an issue for you?
    b. What, if any, factors are important to you in terms of culture, race, ethnicity, gender, or sexual identity when selecting a provider?
25. We mentioned earlier that the MassHealth Accountable Care Organization program started about four years ago. As a reminder, an Accountable Care Organization is a group of healthcare providers that work together as a team to provide care to patients. In what ways, if any, is your care **different now than before, say, two to three years ago**, that are not related to the pandemic?
    a. How is it the same?
    b. What is better? Why?
    c. What may be worse, and why?
26. Is there anything else that you would like to share with us today regarding your healthcare experiences **over the last year**? Have there been any large changes?
27. Overall, has your quality of life changed **in the last year** because of your health or healthcare? If so, how has it changed?
28. Is there anything else you would like to share with us today about MassHealth or your healthcare providers that you haven't already?

[Thank them for their time; Shut off recorder; Confirm any missing demographic information; Collect gift card information]

Gift card vendor: Walmart, Target, or Amazon
Gift card type: electronic or physical

Mailing address:
Email address:
